# Proposal for a new staging system for osteoradionecrosis of the mandible

**DOI:** 10.4317/medoral.19623

**Published:** 2013-12-07

**Authors:** Kemal H. Karagozoglu, Hannah A. Dekker, Derek Rietveld, Remco de Bree, Engelbert A J M. Schulten, Saara Kantola, Tim Forouzanfar, Isaäc van der Waal

**Affiliations:** 1 VU University Medical Center, Department of Oral and Maxillofacial Surgery/ Oral Pathology and Academic Centre of Dentistry Amsterdam (ACTA), Amsterdam, the Netherlands; 2 Vrije Universiteit VU medical center, Department of Radiotherapy; 3 Vrije Universiteit VU medical center, Department of Otolaryngology and Head and Neck Surgery; 4 Oulu University Hospital, Department of Oral and Maxillofacial Diseases, Oulu, Finland

## Abstract

A new staging system for osteoradionecrosis of the mandible has been retrospectively applied to a group of 31 patients. In this system clinicoradiographic signs and symptoms are incorporated in a simplified manner. For imaging purposes the use of plain radiographs such as periapical films and panoramic radiographs is recommended, mainly because of their readily availability. The presented staging system seems well reproducible, facilitating the comparison of study groups dealing with the various issues of osteoradionecrosis of the mandible. It is yet to be evaluated whether the presently proposed staging system is useful for management purposes.

** Key words:**Osteoradionecrosis, jaw bones, mandible, staging, classification.

## Introduction

Osteoradionecrosis (ORN) of the jaws is a relatively rare but serious complication in patients who have been irradiated in the head-and-neck region. Its onset is usually after the first year of irradiation. There are several hypotheses on the etiology and pathogenesis of ORN. Since ORN rarely arises in the maxilla most studies are related to ORN of the mandible. Management is cumbersome and varies from a conservative approach with or without the use of antibiotics and/or hyperbaric oxygen (HbO) treatment to surgery. In an attempt to prevent the disease much attention is paid to elimination of potential odontogenic inflammatory foci prior to radiotherapy and, in case of surgical dental procedures after radiotherapy, to the prophylactic use of antibiotics and peri-operative HbO treatment.

ORN has been defined in various ways. Wong *et al*. defined ORN as “a slow-healing radiation induced ischemic necrosis of bone with associated soft tissue necrosis of variable extent occurring in the absence of local primary tumour necrosis, recurrence or metastatic disease” (1). Chranovic et al. suggested to add to this definition a minimum period of bone exposure of three months (2). Store and Boyson defined ORN as “radiological evidence of bone necrosis within the radiation field, where tumour recurrence has been excluded” (3). The National Cancer Institute defined ORN as “a disorder characterized by a necrotic process occurring in the bone of the mandible”.

In most of the reported cases of ORN no clear diagnostic criteria have been mentioned. Instead, the diagnostic criteria of ORN have often more or less been included in a staging system. In fact, numerous attempts have been made to stage the disease (2,4-10).

The aim of the present study is to evaluate the value of a new staging system, based on a combination of 1) clinicoradiographic findings, and 2) symptoms, such as pain and presence or absence of oral and/or cutaneous fistulas, in patients suffering from ORN of the mandible.

## Patient and Methods

For the purpose of this study ORN has been defined as “radiation induced necrosis of bone”. A diagnosis of ORN was rendered in the presence of exposed bone, with or without changes on a plain radiograph such as a periapical film or panoramic radiograph, having excluded the presence of tumour tissue, either being a second primary or a recurrence. For the purpose of this study a definitive diagnosis of ORN has been made in case of presence of exposed bone for at least one month.

In the period 2005-2010 a total of 50 consecutive patients with ORN of the mandible have been registered in the database of the VU University Medical Center in Amsterdam, the Netherlands and retrospectively evaluated. Nineteen patients were excluded because of recurrent disease (n=10) or incomplete clinical and radiographic documentation (n=9). As a result 31 patients were included, 27 men and four women. The age at the time of the diagnosis of ORN varied from 42 years to 83 years, the mean age being 63 years.

Management of the patients with ORN ranged from observation, the use of various types of antibiotics prescribed in various dosages and routes of administration (orally or systemically), and various surgical procedures with or without peri-operative HbO treatment. In dental implant surgery in patients who had previously received more than 50 Gy irradiation, peri-operative HbO treatment was routinely given (20 sessions preoperatively and 10 sessions postoperatively). Since the focus of this study is on the evaluation of a new staging system of ORN rather than on reporting treatment results, no detailed data on treatment modalities will be provided.

End-points of the staging procedure were 1) clinically resolved condition for a duration of at least one year, 2) segmental resection, 3) death, or 4) end of follow-up.

The staging system is depicted in  Table 1. In this system clinicoradiographic signs and symptoms are incorporated. Staging has been performed at baseline and at various time intervals after the diagnosis of ORN has been established. In case of doubt between two stages the lower stage has been allotted.

**Table 1:**
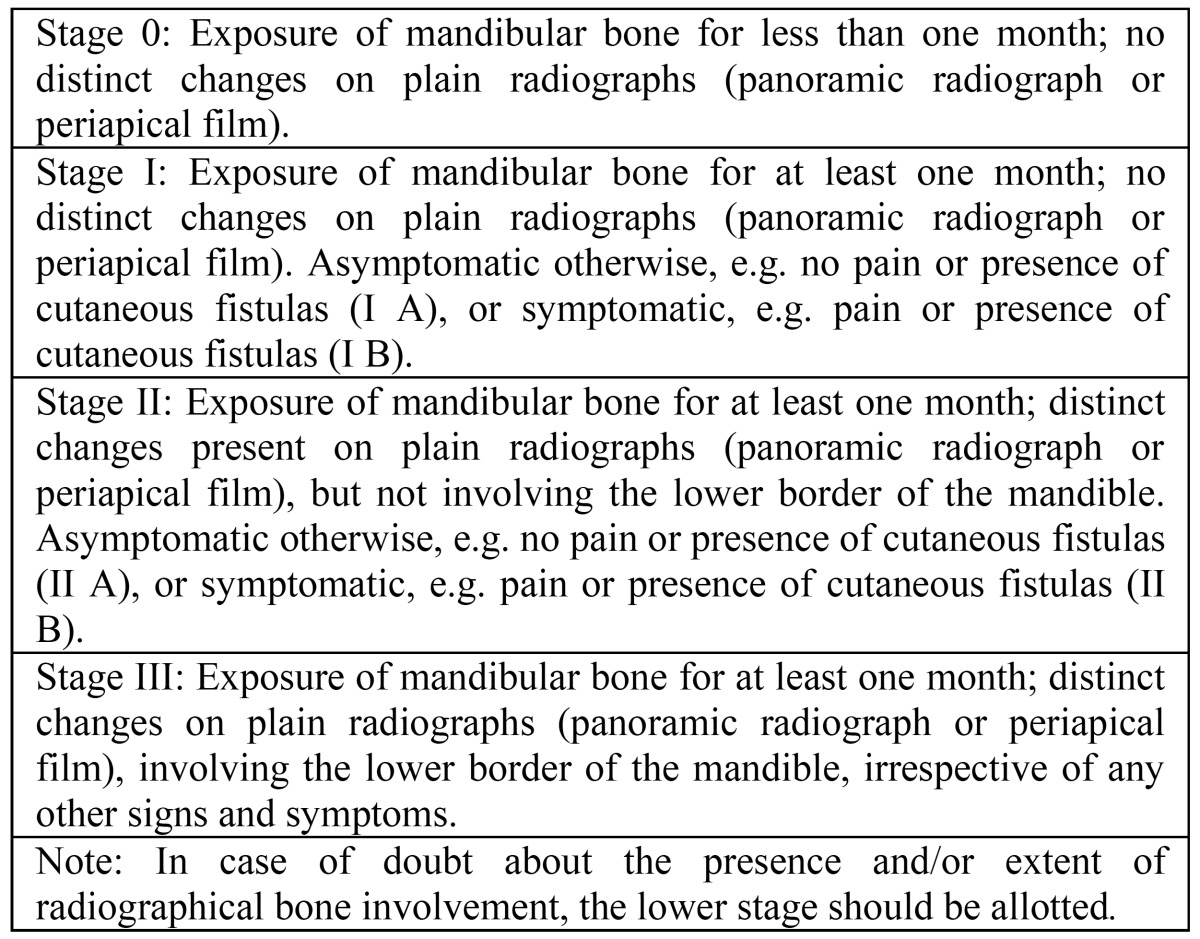
Staging system for osteoradionecrosis of the mandible.

The design of this retrospective study adheres to the code for proper use of human material of the Dutch Federation of Biomedical Scientific Societies (http://www.federa.org).

## Results

**Figure 1:**
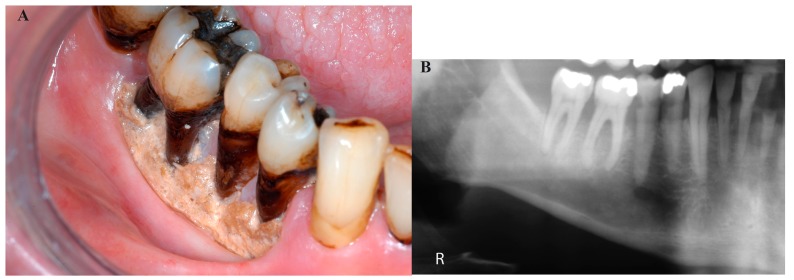
Stage I; exposure of mandibular bone (A); no distinct radiographic changes on the panoramic view (B).

**Figure 2:**
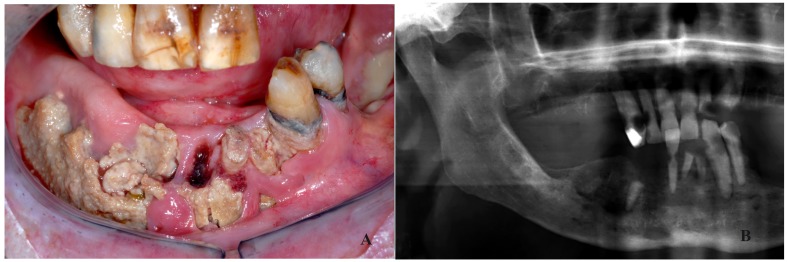
Stage II; exposure of mandibular bone (A); distinct involvement of the mandibular bone but not extending into the lower border (B).

**Figure 3:**
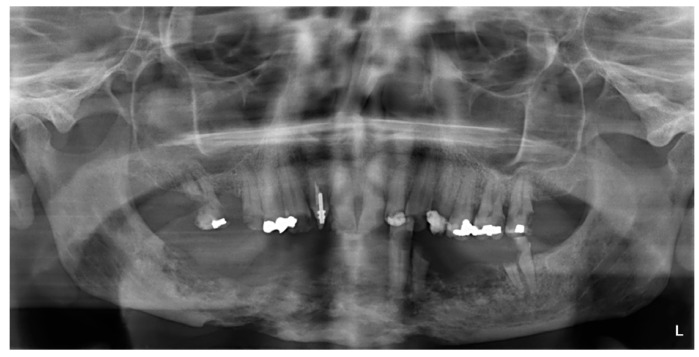
Stage III; extensive osteoradionecrosis of the mandible involving the lower border.

The results of the staging procedure are depicted in  Table 2. Twelve patients were staged as stage I, showing exposure of mandibular bone without distinct changes on plain radiographs (Fig. 1), 15 patients were staged as stage II, showing distinct radiographic changes on plain radiographs but not extending into the lower border of the mandible (Fig. 2), and five patients were staged as stage III at the time of the diagnosis of ORN, showing radiographic involvement of the lower border of the mandible (Fig. 3).

**Table 2:**
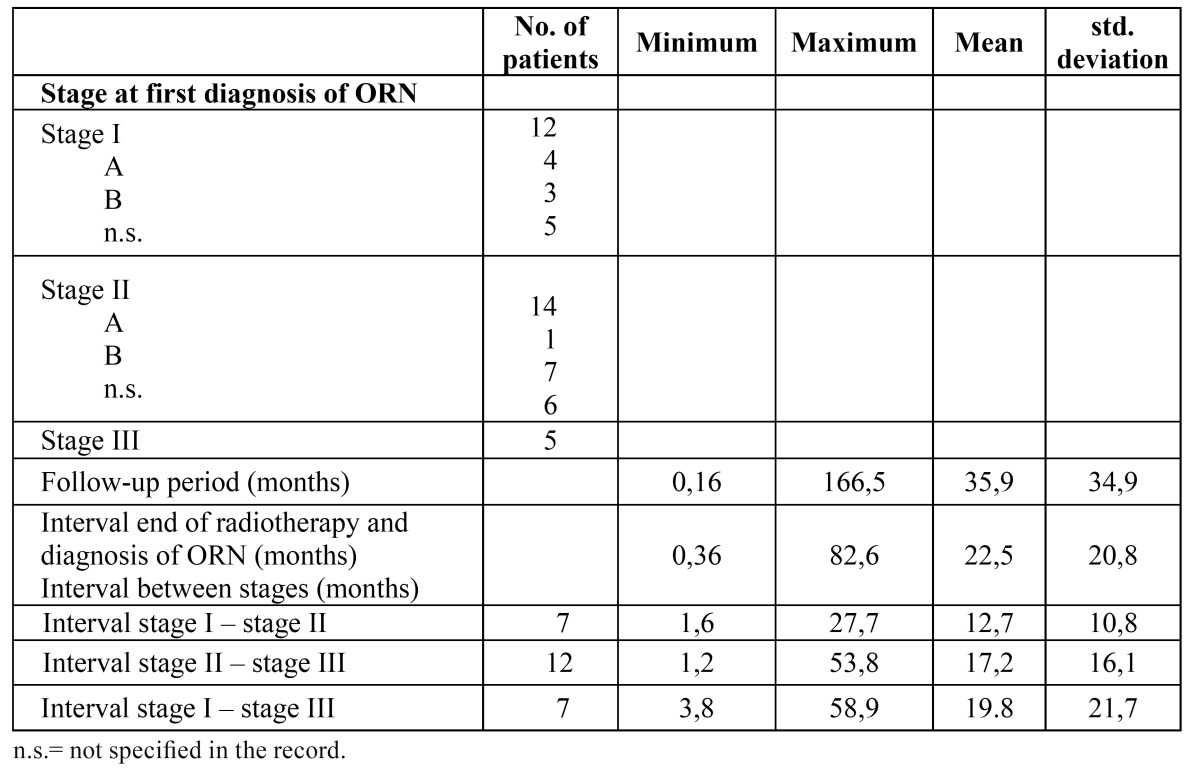
Stage at first diagnosis of osteoradionecrosis (ORN) and during follow-up of 31. Patients.

The mean follow-up period of the entire group of patients was almost 36 months, while the mean interval between stage I and stage II and between stage II and stage III amounted 12.7 months and 17,2 months, respectively. In seven patients, evolving directly from stage I to stage III, apparently no radiographs at stage II had been made. In these cases the mean interval between stage I and stage III amounted 19,8 months.

In  Table 3 the reasons for end of follow-up are shown.

**Table 3:**
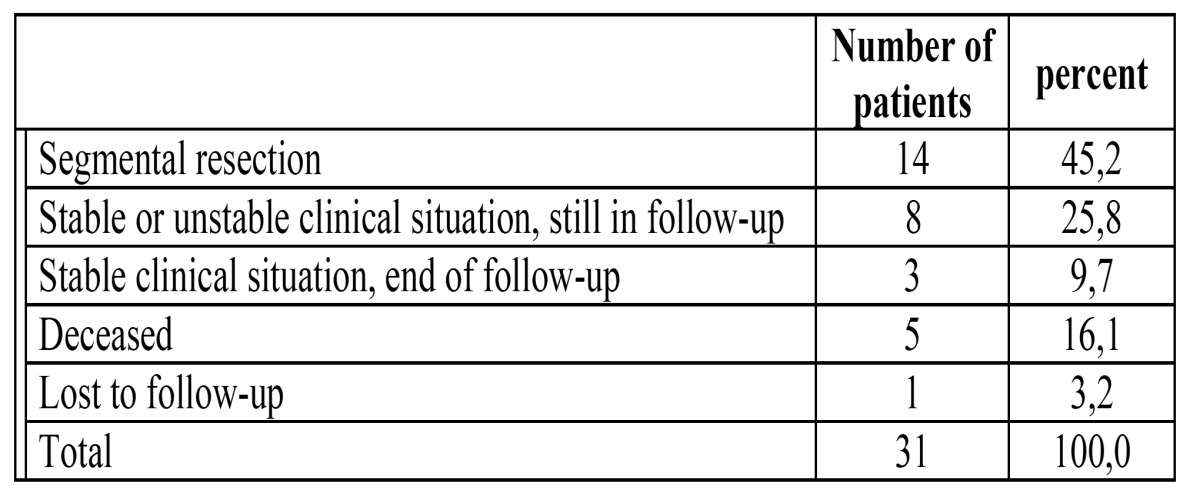
Reasons for end of follow-up in patients with osteoradionecrosis (n=31).

## Discussion and Conclusions

For reporting purposes it seems advisable to observe a minimum time period of bone exposure, e.g. one month, before applying a definitive diagnosis of ORN in the absence of radiographic changes. Within the period of the first month the diagnosis ORN can then be regarded as a tentative, provisional one. However, when radiographic changes are observed already at the first visit of the patient, there is no need to observe such a period of one month before applying a definitive diagnosis of ORN.

In rare cases there may be distinct radiographical bone destruction (stages II and III) during follow-up in the absence of clinically exposed mandibular bone, being asymptomatic otherwise. In the present study these patients have been labeled as having stable disease ( Table 3). In four patients the mandibular bone exposure had resolved while the radiographic changes remained present to some extent.

It is well appreciated that conventional radiographic imaging, such as orthopantomography, may result in an underestimation of the extent or severity of ORN. Interpretation of possible changes in the midline of the mandible can be difficult at times. Also the presence of dental implants may hamper proper evaluation of possible alterations of the surrounding bone. Furthermore, there may be a considerable intraobserver and interobserver variation in the evaluation of periapical films and panoramic radiographs. To some extent, these possible biases may be minimized by the recommendation to downstage the interpretation of the radiographic findings in case of doubt.

In spite of possibly more informative imaging tools such as CT scanning, positron emission tomography (PET), MRI, and radionuclide bone scanning, a panoramic radiograph and/or a periapical film is at present still the most readily available imaging tool worldwide.

In the paper by Chrcanovic *et al*. an excellent overview is given of the various staging systems of ORN that have been proposed in the past (2). Their criticism is that some of these systems are based on response to treatment and, therefore, can only be used retrospectively. It seems preferable, indeed, to use a system that can be used prospectively as was done in the present study. Our staging system seems well reproducible, facilitating the comparison of study groups dealing with the various issues of ORN of the mandible. It is yet to be evaluated whether this staging system is valuable for management purposes.
